# The phylogeographic history of *Krascheninnikovia* reflects the development of dry steppes and semi-deserts in Eurasia

**DOI:** 10.1038/s41598-021-85735-z

**Published:** 2021-03-23

**Authors:** Anna Seidl, Karin Tremetsberger, Simon Pfanzelt, Frank R. Blattner, Barbara Neuffer, Nikolai Friesen, Herbert Hurka, Alexander Shmakov, Oyuntsetseg Batlai, Anže Žerdoner Čalasan, Polina V. Vesselova, Karl-Georg Bernhardt

**Affiliations:** 1 Department of Integrative Biology and Biodiversity Research, Institute of Botany, University of Natural Resources and Life Sciences, Vienna (BOKU), Gregor-Mendel-Straße 33, 1180 Vienna, Austria; 2 Experimental Taxonomy, Leibniz Institute of Plant Genetics and Crop Plant Research (IPK), Corrensstraße 3, 06466 Gatersleben, Germany; 3 School of Biology/Chemistry, Osnabrück University, Barbarastraße 11, 49076 Osnabrück, Germany; 4 Botanical Garden of the Osnabrück University, Albrechtstraße 29, 49076 Osnabrück, Germany; 5 Department of Pharmaceutical and Natural Sciences, I.M. Sechenov First Moscow State Medical University Ministry of Health of the Russian Federation, Izmailovsky Boulevard, 8, Moscow, 105043 Russia; 6 South Siberian Botanical Garden, Altai State University, Lenina 61, 656049 Barnaul, Russia; 7 Department of Biology, School of Arts and Science, National University of Mongolia, University street 3, 14201 Ulaanbaatar, Mongolia; 8 Institute of Botany and Phytointroduction, Committee of Forestry and Wildlife, Ministry of Ecology, Geology and Natural Resources of the Republic of Kazakhstan, Timiryazeva Street 36D, 050040 Almaty, Kazakhstan

**Keywords:** Ecology, Biodiversity, Biogeography, Grassland ecology, Molecular ecology

## Abstract

Constituting one of Earth’s major biomes, steppes are characterised by naturally treeless extra-tropical vegetation. The formation of the Eurasian steppe belt, the largest steppe region in the world, began in Central Asia during the Neogene. In the glacial stages of the Pleistocene, steppe displaced forest vegetation, which in turn recolonised the area during the warmer interglacial periods, thus affecting the distribution of plants adapted to these habitats. *Krascheninnikovia ceratoides* (Chenopodiaceae) is a plant characteristic of dry steppe and semi-desert formations. Earlier studies showed that the ancestor of this autochthonous steppe element originated in Central Asia during the Miocene/Pliocene, i.e., in the same region and at the same time as the first appearance of steppe vegetation. However, as the extant lineages of *Krascheninnikovia ceratoides* diversified only 2.2 ± 0.9 Mya, it may represent a modern element of current dry steppe and semi-desert formations, rather than a component of the first steppe precursors of the Miocene. As such, it may have capitalised on the climatic conditions of the cold stages of the Quaternary to expand its range and colonise suitable habitats outside of its area of origin. To test this hypothesis, phylogeographic methods were applied to high-resolution genotyping-by-sequencing data. Our results indicate that *Krascheninnikovia* originated in western Central Asia and the Russian Altai, then spread to Europe in the West, and reached North America in the East. The populations of eastern Central Asia and North America belong to the same clade and are genetically clearly distinct from the Euro-Siberian populations. Among the populations west of the Altai Mountains, the European populations are genetically distinct from all others, which could be the result of the separation of populations east and west of the Urals caused by the Pleistocene transgressions of the Caspian Sea.

## Introduction

Steppes are characterised as naturally treeless extra-tropical vegetation dominated by drought-resistant xeromorphic grasses, perennial herbs, and low shrubs, geophytes and therophytes^1–3^. The Eurasian steppe belt is the largest steppe region in the world, stretching from the Hungarian basin in the West to the Amur River in the East. Its formation presumably started in Central Asia in the Neogene^1^ [and literature cited therein]. Significant Northern Hemispheric glaciation started at the beginning of the Pleistocene. In the last 2.6 Million years (My), numerous cold-warm cycles have occurred, as indicated by oxygen isotope studies of marine sediments^4^. During the cool glacial periods, steppes spread at the expense of forests, while forests recolonised the same areas in the warmer interglacial periods^5^. The distribution range of plants growing in steppe and semi-desert formations has expanded and contracted accordingly^6^. Some important landscape structures that may have affected the ability of steppe plants to migrate within their appropriate habitat during the ice ages were permanent, such as the Altai Mountains in Russia and Mongolia, or the Khangai Mountains of Central Mongolia^7^. On the other hand, temporarily large bodies of water in the area south of the Ural Mountains, caused by transgressions of the Caspian Sea during the ice ages, may have inhibited the migration of steppe plants for only a limited time^1, 8–10^.

In Central Europe, the remains of the more or less continuous Pleistocene *Artemisia*-Chenopodiaceae cold steppe (tundra-steppe) belt^1, 5^ can be found nowadays only in azonal steppe islands and in artificial, man-made steppe-like habitats^11, 12^. In recent years, steppe plants and their shared history with the Eurasian steppe belt have come under the focus of much research^13–21^.

**Figure 1:**
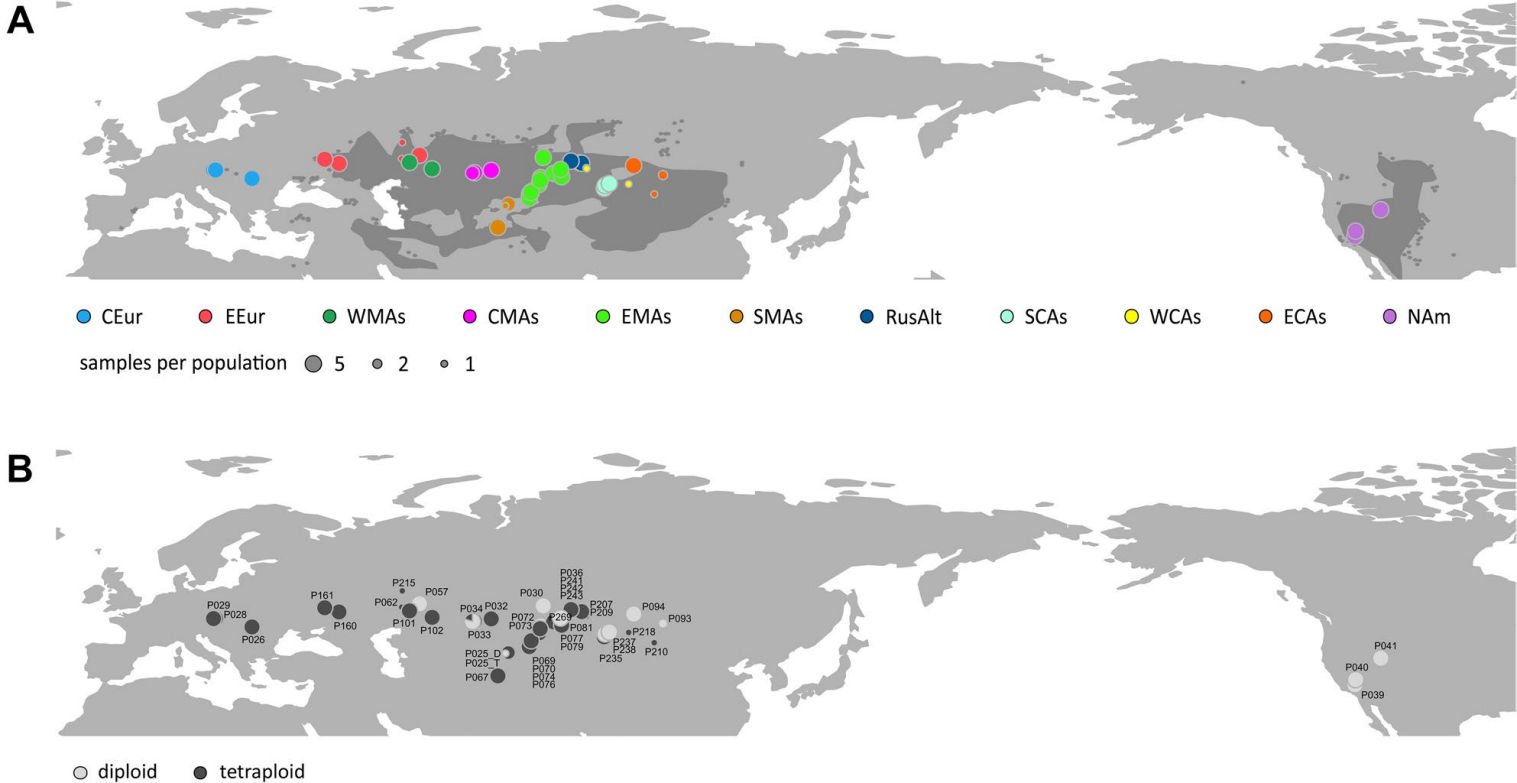
Population sampling of *Krascheninnikovia ceratoides*. Up to five individuals per population were used, as indicated by dot size. (**A**) The colour coding indicates the assigned geographical region of the population following Brummit (2001) (*CEur* Central Europe, *EEur* eastern Europe, *WMAs* west Middle Asia, *CMAs* central Middle Asia, *EMAs* east Middle Asia, *SMAs* south Middle Asia, *RusAlt* Russian Altai, *SCAs* south Central Asia, *WCAs* western Central Asia, *ECAs* east Central Asia, *NAm* North America). The shaded area indicates the distribution area of *K. ceratoides*. (**B**) The ploidy is indicated by colour: diploid (grey) and tetraploid (black). The map was generated in R using the package ‘rworldmap’ (http://journal.r-project.org/archive/2011-1/RJournal_2011-1_South.pdf) and edited in Inkscape v1.0.2 (https://inkscape.org).

We still know little about where and when the constituent taxa of the various steppe and semi-desert formations originated, and how they have spread. Due to its distribution and age, the xeromorphic shrub species *Krascheninnikovia ceratoides* (L.) Gueldenst. (Chenopodiaceae) is a particularly suitable subject for reconstructing the evolutionary history of dry steppe and semi-desert formations. It is widespread throughout the Northern Hemisphere, growing on shortgrass steppes, desert steppes, and in semi-deserts^22, 23^. Although its high morphological variation has led to the recognition of a variety of taxonomical names, *Krascheninnikovia* is now thought to constitute a single species with two subspecies, the Eurasian *K. ceratoides* subsp. *ceratoides* and the North American *K. ceratoides* subsp. *lanata*^23^. The distribution of *K. ceratoides* subsp. *ceratoides* is broad and continuous, extending from Mongolia and northern China in the East to the southern Volga and Don regions (European Russia) in the West (Fig. 1). To the South, it reaches the Himalayas and Iran. Large parts of its distribution overlap the Irano-Turanian floristic region^24, 25^. In Europe, small and isolated populations occur in xerothermic grasslands and on the dry steppes of Austria, Romania, Spain, and Ukraine. *Krascheninnikovia ceratoides* subsp. *lanata* is present on the shortgrass prairies, dry scrubland, and in semi-desert regions of central and western Canada, the USA, and Mexico^22^.

*Krascheninnikovia ceratoides* is a long-lived shrub growing 0.1 to 2 m tall, depending on the habitat^26^. It can be characterised as an eurythermal species^1^ that can withstand a wide range of hot and cold temperatures, and grows from sea level (e.g. in the lowlands of Kazakhstan) to high elevations of 4500–4800 m in the southern part of its range, such as in the Pamir and Ladakh mountain ranges^26, 27^. In Asia, the plant is used for firewood and fodder, with its overuse leading to shrinking population sizes and concomitant possible land degradation as a consequence of increased erosion^28^. This phenomenon is called the *Teresken* syndrome for Teresken, the common name for *K. ceratoides* in Russian^28, 29^. In North America it is used as fodder for livestock in winter (‘winterfat’)^30, 31^. *Krascheninnikovia ceratoides* plants can reach an age of 300 years^32^, and first reproduction only occurs in plants older than 25 years^33^. The species is believed to be outcrossing^34^. The bractlets surrounding its fruit and utricle are densely covered with long hairs, which may facilitate dispersal by wind.

Various ploidy levels exist in *K. ceratoides* throughout its distribution range. Diploids with 2*n* = 18 chromosomes have been reported from the Ural Mountains of western Russia, across Kazakhstan and Kyrgyzstan to Mongolia and China^35–40^. Tetra- and/or hexaploids (2*n* = 36 and 54) have been reported from Europe to the Russian Altai Mountains^35, 41–46^, and south of this range in Armenia^47^, China^36, 38, 39^, Iran^48^, Kyrgyzstan^37^, and Tajikistan^40^. A mixed population of di-, tri-, tetra-, penta-, and hexaploids (2*n* = 18, 27, 36, 45 and 54) has been reported from Kyrgyzstan^37^. Diploid and tetraploid populations occur in North America^35, 49^.

According to previous studies, the stem group of *Krascheninnikovia* originated c. 5 to 23 Million years ago (Mya) (~ 21.2 Mya^50^; 12.8 Mya (5.3‒23.1 Mya)^51^; 17.3 Mya (12.0‒23.5 Mya)^35^), while the crown group diversified 2.2 ± 0.9 Mya^35^. With a minimum age of more than 5 My, *K. ceratoides* has persisted throughout several global climatic changes including the glacial and interglacial phases of the Quaternary. Its diversification into extant lineages took place during the Gelasian (Early Pleistocene, 2.6‒1.8 Mya), and seemingly correlates with the appearance of modern types of desert and semi-desert. These are, together with semi-arid temperate grasslands such as prairie, steppe, and pampas, a Plio-/Pleistocene phenomenon^52^. Therefore, *K. ceratoides* could have been among the first plants that inhabited modern-type dry steppe areas.

Seidl et al.^35^ reconstructed the biogeographical history of *Krascheninnikovia* using a few molecular markers from its nuclear and chloroplast genomes (ITS, ETS, *atp*B*-rbc*L, *rpl*32*-trn*L, *trn*L*-trn*F). Genetic analyses relying on single loci may reflect only the history of these particular loci, rather than the history of the species studied. Therefore, a whole-genome approach is favourable, where single loci have less weight and cannot influence the result as strongly. To investigate the spatio-temporal diversification of *K. ceratoides* in more detail, we sequenced populations of the species from throughout most of its distribution range employing genotyping-by-sequencing (GBS). We then inferred phylogenetic trees and networks from the GBS data, conducted ancestral range reconstructions, and measured genome size. We specifically addressed the following questions: (1) Where did *Krascheninnikovia* originate? (2) Can GBS provide the appropriate resolution to retrace its colonising history and migration routes? (3) Can the retrieved phylogeographic and population genetic patterns be related to palaeogeographical and palaeoenvironmental events during the evolutionary history of the Eurasian steppe? With this, our overall aim was to contribute to the knowledge of the florogenesis of the Eurasian steppe belt.

## Materials and methods

### Plant material

*Krascheninnikovia ceratoides* leaves were collected in the field and dried with silica gel or taken from herbarium material. Leaves from up to five individuals per population were collected. The five individual specimens from Kyrgyzstan combined into population P025 came from two different localities about 80 km apart. In total, 183 accessions from 43 different populations of *K. ceratoides* were used for GBS analyses (Fig. 1; Supplementary Table S1). Most of the samples were the same as those used in a previous phylogenetic study of ITS, ETS, and chloroplast DNA sequences^35^. Three North American populations determined as *K. ceratoides* subsp. *lanata* (P039, P040, P041) were included, as well as two eastern Middle Asian populations of *K. ceratoides* subsp. *ceratoides*, which were originally determined as *K. ewersmanniana* (P069, P070). *Axyris hybrida* and *Ceratocarpus arenarius* were used as outgroups. Further details to the collected samples can be found in Supplementary Table S1.

The present study complies with relevant institutional, national, and international guidelines and legislation.

### Genome size measurements

We determined the genome size and ploidy level of 99 samples of *K. ceratoides* leaf tissue by flow cytometry, using an internal standard (either *Pisum sativum* L. ‘Kleine Rheinländerin’ (2C = 8.8 pg^53^) or *Petroselinum crispum* (Mill.) Fuss (2C = 4.5 pg^54^)) and the CyStain PI Absolute P kit (Sysmex, Görlitz, Germany) according to the manufacturer’s instructions. The relative fluorescence of at least 5000 particles per sample was recorded with a CyFlow Space (532 nm diode laser; Sysmex).

### DNA extraction and GBS

The accessions used in the study by Seidl et al.^35^ were complemented with additional individuals from both the same and new populations (P076, P079, P102, P207, P241, P269; Supplementary Table S1). Genomic DNA was extracted from about 20 mg dried leaf material using the DNeasy Plant Mini Kit (Qiagen, Hilden, Germany), the NucleoSpin Plant II kit (Macherey–Nagel, Düren, Germany), or the innuPREP Plant DNA Kit with the SLS CTAB-containing lysis solution (Analytic Jena, Jena, Germany) according to the manufacturers’ instructions. Five DNA samples were extracted from herbarium leaf material (P207‒P218). DNA concentrations were quantified with a DS-11 FX Fluorometer (DeNovix, Wilmington, Delaware, USA), using the DeNovix dsDNA Broad Range Kit (DeNovix) according to the manufacturer’s manual.

Genotyping-by-sequencing library preparation followed Poland et al.^55^, using the restriction enzymes *Msp*I and *Pst*I. Samples were sequenced in two runs on a HiSeq 2500 (Illumina, California, USA) (single-end sequencing of 100 bp fragments). To achieve the same genomic coverage for individuals with different ploidies, twice the amount of DNA was used for tetraploids as for diploids. Two individuals (one from population P029 and one from population P072) were sequenced twice to assess the reproducibility of the method.

### Data analysis

We used STACKS v.2.55^56^ for the sorting, filtering and de novo assembly of raw read data. To find the optimal parameter settings, we ran STACKS ‘denovo_map’ on a reduced data set (Supplementary Methods S2). We varied one of the three following parameters while keeping the others at their default value: the minimal number of identical reads required per stack, m, was tested in a range from three to six; the differences between loci within an individual, M, was tested for one to four base pair differences; the differences allowed between catalog loci, n, was tested for one to eight base pair differences. The parameter value that maximized the number of variant loci present in at least 80% of all individuals were used for the complete data set: m = 3, M = 3 and n = 8. We set the number of stacks per locus to five to account for possibly present allelic variation in tetraploids. Diploid and tetraploid samples were treated identically. The STACKS ‘population’ program using the ‘write random SNPs’ flag was used to produce unlinked SNP data sets, with and without the *Axyris* and *Ceratocarpus* outgroups.

To estimate ploidy levels from GBS data, we used nQuire^57^ and ploidyNGS^58^, which recognize bam-files as input. These were built using BWA (Burrows-Wheeler Alignment tool)^59^ with the concatenated consensus sequence of the respective individuals serving as a reference. BWA settings were the following: maximum gap length of twelve, maximum of one gap per read, and a mismatch parameter of 0.01^60^. These bam-files were used as input for nQuire using the denoising option and for ploidyNGS using the “guess ploidy” option.

To infer the population genetic structure of *Krascheninnikovia*, an analysis was performed in R^61^ using the LEA^62^ package, using the data set of unlinked SNPs in Variant Call Format (VCF) of ingroup samples only. The ploidy was set to tetraploid. One to fifteen hypothetical ancestral populations were tested with 100 repetitions each. The number of clusters was chosen based on minimal cross-entropy. To show the result per population, the affiliation to each hypothetical ancestral population was calculated per population as the mean over its individuals and shown as pie charts on a map. To validate the results using a different approach, both Discriminant Analysis of Principal Components (DAPC) and Principle Coordinate Analysis (PCoA) were performed using the R packages^63, 64^ adegenet and dartR, respectively, based on the ingroup data set of unlinked SNPs.

Unlinked SNPs of 6,029 loci were retained in the PHYLIP file of *Krascheninnikovia* as consensus sequences per population, after removing all uninformative sites^65^. Phylogenetic analyses were conducted by applying the maximum likelihood (ML) criterion using^66^ IQ-TREE v.2.0.3^67^ with the GTR+G model and correction for ascertainment bias^68^. SH-like approximate likelihood ratio test^69^ and ultrafast bootstrapping (UFBoot)^70^ were performed with 1,000 repetitions each to obtain bootstrap support (aLRT/UFBoot BS) for nodes. The outgroups *Axyris* and *Ceratocarpus* are genetically too distant to assess the correct rooting, so midpoint rooting was used, which resulted in a tree with basal positions for populations of the Russian Altai. The basal population of the IQ-TREE analysis in Seidl et al.^35^, P242, originated in the Russian Altai as well. The splits data was visualised in SplitsTree v4.14.8^71^ and the consensus tree in FigTree v1.4.3^72^.

To calculate private allelic richness per region, a rarefaction analysis was performed in HP-Rare^73^. Populations were assigned to biogeographic regions according to their origin based on Brummit^74^: Central Europe, eastern Europe, western Middle Asia, central Middle Asia, eastern Middle Asia, southern Middle Asia, the Russian Altai Mountains, western Central Asia, southern Central Asia, eastern Central Asia, and North America. Only loci with called bases present in all populations were used. Rarefaction sample sizes for each ‘SNP locus’ were two populations from each region, and two alleles (‘genes’ in HP-Rare) from each population. Mean rarefaction and standard deviation were then calculated per region.

### Ancestral range reconstruction

An ultrametric tree was built based on the ML tree using PATHd8^75^ as input tree for the BioGeoBears v. 1.1.1^76–78^ R package for R ver. 3.5.0^76–78^. The assignment of populations to regions was described above. The analysis was performed based on two different migration hypotheses: free migration between all areas, and restricted migration due to the distance between areas (0.8 for regions with a common land border and 0.2 for areas without a common land border). Three different biogeographic models were tested: DEC, DIVALIKE, and BAYAREALIKE, all both with and without the jumping parameter “j”, which determines the probability of jump dispersals. Based on AICc values, DIVALIKE+J with restricted migration was chosen as the most likely model. DIVALIKE is a likelihood interpretation of parsimony-based DIVA and allows the same processes^79^ such as dispersal, extinction, narrow sympatry, narrow and widespread vicariance, but not, e.g., subset sympatry.

## Results

### Ploidy

The mean 2C genome sizes (± standard deviation) of diploid (N = 38) and tetraploid (N = 61) individuals were 2.8 ± 0.3 pg and 5.8 ± 0.2 pg, respectively. The populations measured were uniformly di- or tetraploid. The populations from Central Europe, western Middle Asia, and the Russian Altai Mountains were all tetraploid, but in most regions both diploid and tetraploid populations were found (Fig. 1; Supplementary Table S1). The genome sizes of 85 samples could not be measured due to the old age of the material, their storage conditions (e.g., populations from eastern Europe: P215; central Middle Asia: P034; western Central Asia: P207, P209, P218; eastern Central Asia: P210; Supplementary Table S1), or a lack of material. Only one individual each of the populations P034 (central Middle Asia) and P025 (Kyrgyzstan) could be measured, both turning out to be diploid.

Ploidy level estimations based on GBS data using nQuire and ploidyNGS confirmed most of these measurements. Only the North American sample P041-I0372 was inferred to be tetraploid but appeared diploid according to flow cytometry measurements. Of those not measured using flow cytometry, the populations of eastern Europe (P215), western Central Asia (P207, P209, P218), and eastern Central Asia (P210) were found to be tetraploid; of P025, all individuals were found to be tetraploid except P025-I0223, which was diploid in both analyses, by GBS and flow-cytometry data. Consequently, we consider it very likely that P025-I0223 came from one of the two Kyrgyz localities, and the other four P025 individuals from the other locality, which is ~ 80 km away. P025-I0223 (hereinafter P025_D) and the other four individuals (hereinafter P025_T) were therefore treated as two different populations in all analyses. Of population P034, one individual (P034-I0317) was tetraploid, while all others were diploid. In all populations except for P025 and P034, the GBS-estimated ploidy of unmeasured samples corresponded to the ploidy of measured individuals from the same population.

### Raw data analysis

Of 24,249 loci, composed of 2,625,739 sites, 21,254 variant and unlinked sites remained in the STACKS VCF file of *Krascheninnikovia*. Of those loci, 6029 were kept after removing all uninformative sites. In the PHYLIP file including the outgroups, only 500 SNPs of *Axyris* and 525 SNPs of *Ceratocarpus* were retained, while the populations of *Krascheninnikovia* comprised on average 3731 (± 883) SNPs. Due to this huge difference of the amount of recovered data, the analyses including the outgroups may not be meaningful and were dismissed.

### Genetic clusters

**Figure 2:**
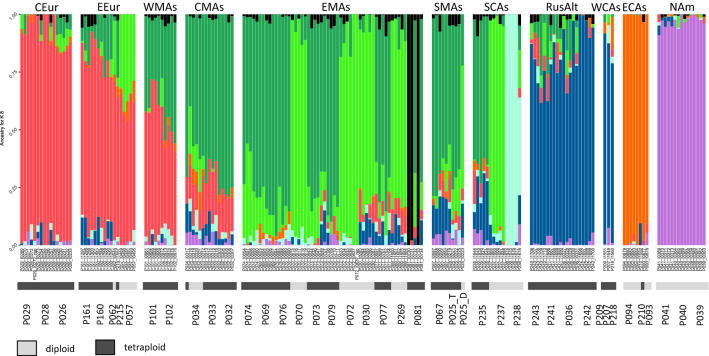
Genetic cluster membership as inferred using LEA is shown as barplot. Vertical bars denote individuals. Eight clusters were retained, which are indicated by different colours. The samples are ordered by their assigned geographical region from west to east. Ploidy is indicated by grey (diploid) and black (tetraploid) bars. The figure was generated in R using the package ‘ggplot2’ (https://ggplot2.tidyverse.org) and edited in Inkscape v1.0.2 (https://inkscape.org).

LEA assigned all individuals to hypothetical ancestral populations based on their genetic composition. After 100 repetitions, eight hypothetical ancestral populations (K) were chosen based on the cross-entropy criterion (Supplementary Figure S3). The red cluster (Fig. 2) is mainly represented by populations from Central Europe, eastern Europe, and western Middle Asia (all west of the Ural Mountains, mostly tetraploids except for populations P057 and P062 from eastern Europe). The dark green cluster mainly contains tetraploid populations from western, central, eastern, and southern Middle Asia, and southern Central Asia. The light green cluster is mainly represented by diploid populations from eastern and southern Middle Asia and from southern Central Asia and is present in the diploids of eastern Europe and central Middle Asia. The black cluster is represented by three individuals of P081 of eastern Middle Asia, the light blue cluster by P238 of southern Central Asia. The dark blue cluster is mainly comprised of populations from the Russian Altai Mountains and western Central Asia. Populations from eastern Central Asia form the orange cluster. The populations from North America belong to the lilac cluster. Some admixture is observed, especially between the light green, dark green, and red clusters (Fig. 2).

**Figure 3:**
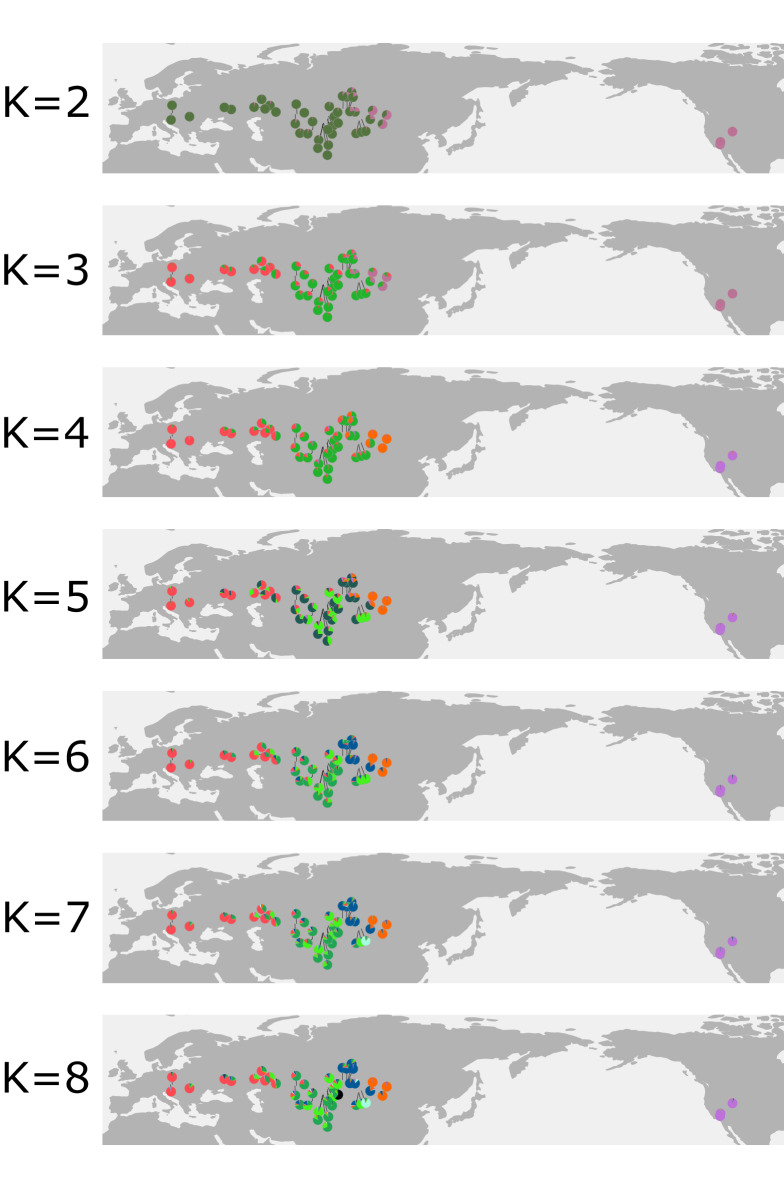
Genetic clustering of samples as inferred from LEA is shown as pie charts for K=2 to K=8 clusters. The first split occurred in Central Asia, dividing the populations west and east of the Khangai Mountains (K=2), followed by a split in the area of the Ural Mountains (K=3). At K=4, the American populations separate; at K=5, the diploids and tetraploids of Middle Asia and adjacent areas each form their own group. At K=6, the populations of the Altai Mountains form their own cluster. At K=7 and K=8, P238 and P081 form their own cluster. The map was generated in R using the packages ‘mapplots’ (https://CRAN.R-project.org/package=mapplots) and ‘rworldmap’ (http://journal.r-project.org/archive/2011-1/RJournal_2011-1_South.pdf) and was edited in Inkscape v1.0.2 (https://inkscape.org).

To illustrate the consecutive splits between the hypothetical ancestral populations, the cluster membership was depicted as pie charts on a map for all K values from two to eight (Fig. 3). The deepest split, with two hypothetical ancestral populations (*K* = 2), was inferred between the populations east of the Khangai Mountains in Central Mongolia (North America and eastern Mongolia), and the populations west of the Khangai Mountains. The second deepest split (*K* = 3) is in the area of the Ural Mountains, dividing the populations west of the Khangai Mountains. With *K* = 4, the Mongolian populations east of the Khangai Mountains and the North American populations each form a group of their own. The populations between the Ural and Khangai Mountains split in two groups according to their ploidy with *K* = 5: diploid populations are assigned to the light green cluster, while tetraploid populations are assigned to the dark green cluster. With *K* = 6, the populations of the Russian Altai and western Central Asia form their own group (dark blue). With *K* = 7, population P238 from southern Central Asia forms its own cluster (light blue), as does P081 with *K*=8 (black).

### DAPC

**Figure 4:**
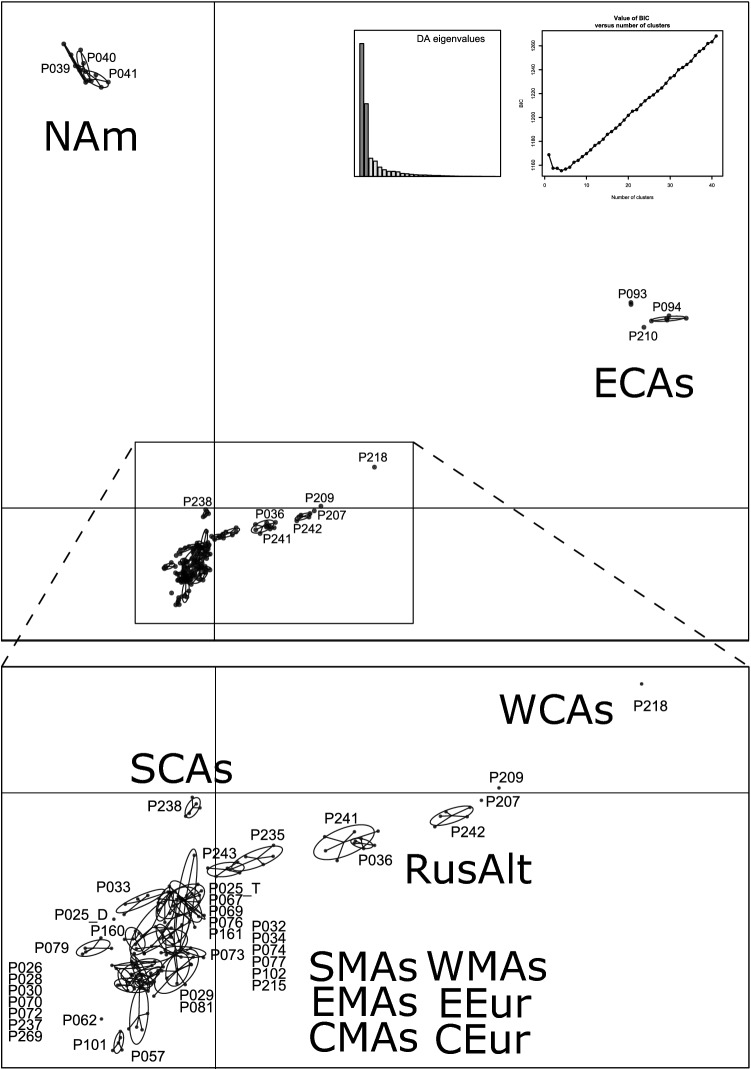
Genetic clustering of samples as calculated by dapc package of R. Four clusters were retained. One cluster comprises the European populations, one the Middle Asian and southern Central Asian populations, one the populations of western Central Asia and the Russian Altai, and another the eastern Central Asian and North American populations. The figure was generated in R and edited in Inkscape v1.0.2 (https://inkscape.org).

Similarly to LEA, DAPC analysis clusters individuals according to their genetic composition (Fig. 4). Four clusters were retrieved: (1) populations from Europe (Ural Mountains to Central Europe, including western Middle Asia), (2) the populations from Middle Asia and southern Central Asia, (3) the populations of the Russian Altai and western Central Asia, and (4) the populations of eastern Central Asia and North America, mirroring the first splits of the analysis performed using the LEA package.

### PCoA

**Figure 5:**
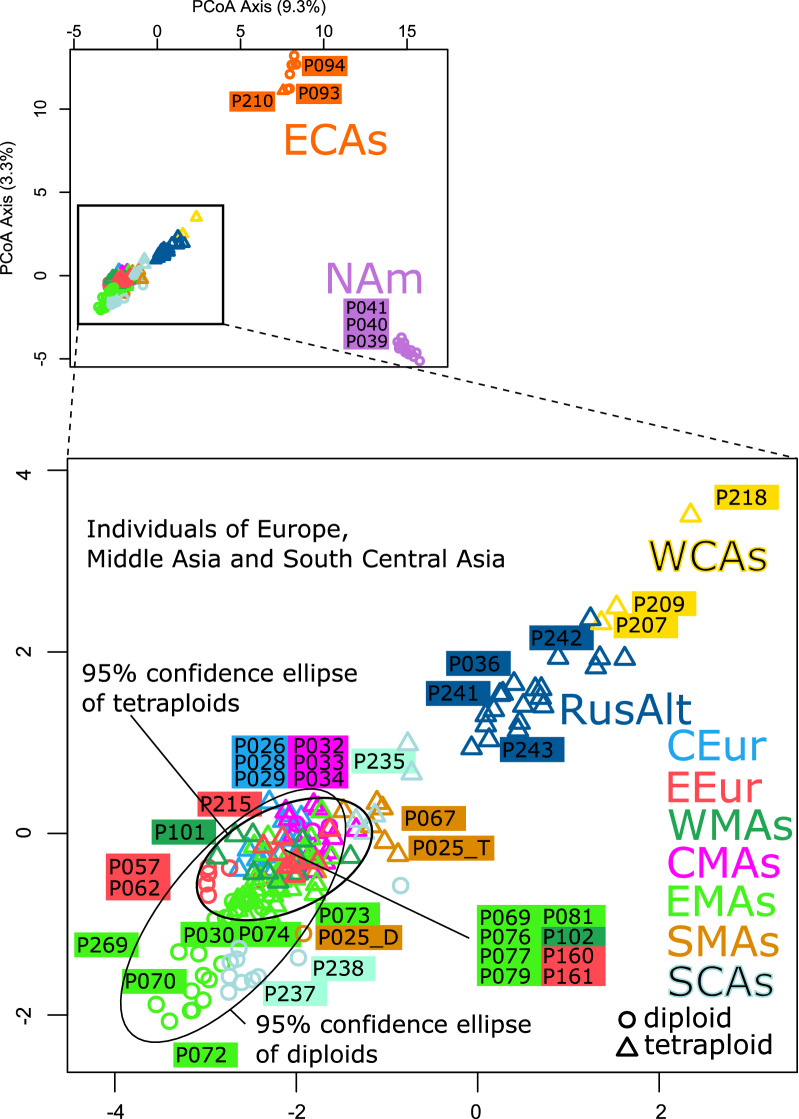
PCoA scatterplot. Symbols representing samples of the same geographical area were coloured according to Fig. 1. Circles indicate diploids, triangle tetraploids. Upper graph: Distinct from most of the samples, the populations of eastern Central Asia and North America each form their own clusters. Lower graph: Within the remaining populations, those from the western Central Asia and from the Russian Altai are genetically distinct. Within the densely scattered other populations, there is a trend visible, which separates diploids and tetraploids from each other. The figure was generated in R using the packages ‘MASS’ (http://www.stats.ox.ac.uk/pub/MASS4/), ‘car’ (https://socialsciences.mcmaster.ca/jfox/Books/Companion/) and ‘graphics’ (https://www.rdocumentation.org/packages/graphics) and was edited in Inkscape v1.0.2 (https://inkscape.org).

Samples from Europe, Middle Asia, southern Central Asia, the Russian Altai and western Central Asia clustered together in the Principal Coordinate Analysis (Fig. 5). Apart from this group, two other clusters were found: samples from eastern Central Asia and samples from North America. The populations of western Central Asia and the Russian Altai are genetically different to other populations of the majority group. Among the samples from Europe, Middle Asia and southern Central Asia, diploids and tetraploids segregated along the second PCoA axis.

### Phylogenetic analysis

**Figure 6:**
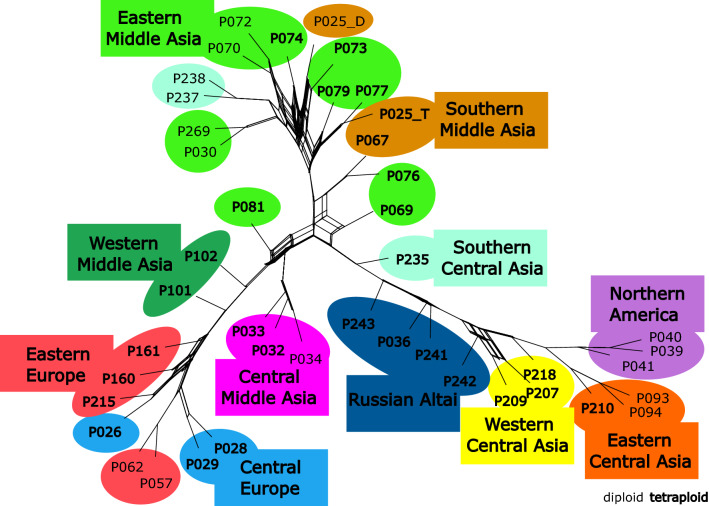
Phylogenetic network based on a ML method (IQ-TREE). The origin of the populations is indicated by colour (compare Fig. 1). Tetraploids are shown in bold. The network was visualized in SplitsTree and edited in Inkscape v1.0.2 (https://inkscape.org).

According to the ML tree, *K. ceratoides* comprises two fully supported major genetic groups: populations from the Russian Altai, eastern Central Asia, and North America form one clade, which is a sister to a clade comprising the remaining accessions from the Russian Altai and from southern Central Asia to Central Europe (Fig. 6, Supplementary Figure S4). Within the first group, two well-supported clades of populations from eastern Central Asia and North America, respectively, derive from populations of western Central Asia and one population of the Russian Altai (99.9 aLRT/100 UFBoot BS). In the second group, populations from the Russian Altai Mountains constitute the basally branching lineages, forming a grade from which the southern Central Asian, Middle Asian and European populations are derived (Supplementary Figure S4). The populations of central Middle Asia form a well-supported clade (98.8/99), as do the populations west of the Ural Mountains (63.2/94, excluding P102: 93.6/98). In this clade, populations P101 and P102 from western Middle Asia are paraphyletic sister groups to the European clade. The replicates of the two individuals for which GBS analysis was performed twice (P029-I0272, P072-I0637), occur next to each other in the IQ-TREE analysis (100/100), when using data per individual, confirming the reproducibility of the method (data not shown).

### Biogeographic analysis with BioGeoBears

**Figure 7:**
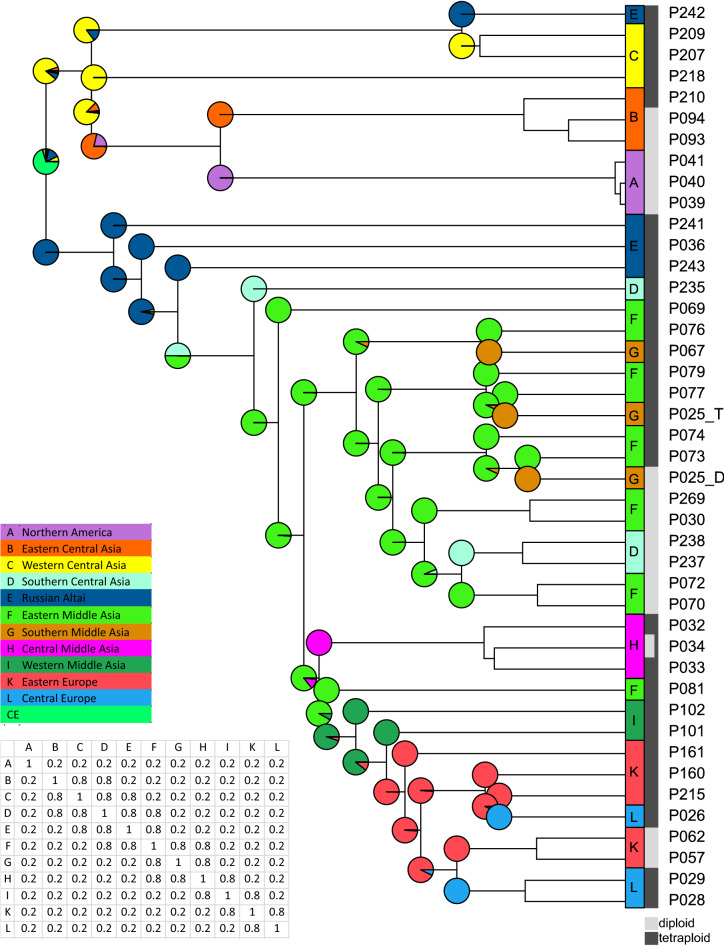
Phylogeographic analysis using the DIVALIKE model of the R package BioGeoBEARS. Colours indicate the origin of the samples, compare Fig. 1. Branches were collapsed when several members of the same populations clustered together. *Krascheninnikovia* spread from the Altai Mountains to the East, reaching eastern Central Asia and North America, and to the West, reaching Middle Asia and Europe. The figure was generated in R and edited in Inkscape v1.0.2 (https://inkscape.org).

Based on our BioGeoBEARS analysis, the ancestral range of *K. ceratoides* lies within the area of western Central Asia (Mongolian Altai Mountains, yellow) and the Russian Altai Mountains (dark blue) (Fig. 7). From western Central Asia, *K. ceratoides* migrated east to eastern Central Asia (orange) and from there to North America (lilac). From the Russian Altai Mountains, it migrated to southern Central Asia and to eastern Middle Asia (light green). From eastern Middle Asia, it then migrated again to southern Central Asia (light blue) and to southern Middle Asia (brown). It migrated further west to central Middle Asia (pink) and to western Middle Asia (dark green), and on to eastern Europe (red) and Central Europe (blue).

### Private allelic richness

The highest private allelic richness was found in western Central Asia (0.014 ± 0.002), followed by eastern Central Asia (0.013 ± 0.004) and the Russian Altai Mountains (0.011 ± 0.001; Supplementary Figure S5). The lowest private allelic richness was found in North America (0.007 ± 0.003).

## Discussion

In this study we reconstructed the phylogeographic history of *K. ceratoides*, a widespread and representative steppe inhabitant, to gain insights into the general evolutionary history of the Eurasian steppe belt. The retrieved molecular patterns fit best to a scenario of a western Central Asian/Russian Altai origin of *Krascheninnikovia* and its subsequent spread both towards the West, through stepwise migration until reaching Central Europe, and towards the East, finally colonizing western North America.

Steppe precursors originated in Central Asia during the Miocene at the latest^1, 80, 81^. From there, the steppe started to spread, with many latitudinal range shifts due to changing climatic conditions^6, 82–85^. A continuous Eurasian steppe belt was first present during the late Miocene/early Pliocene^1^. At the end of the Pliocene, true steppes were present in Central Asia, while forest-steppe assemblies were present in south-eastern Europe and on the West Siberian Plain^5^. Cold steppe vegetation spread during the glacial periods of the Pleistocene^1^. Eurythermal species could have survived the last glacial maximum *in situ*^1^. *Krascheninnikovia ceratoides* is an autochthonous steppe element, which originated in the steppe belt similarly to some species of the genus *Allium*^86^. In contrast, species of the genus *Camelina* migrated into the Eurasian steppe secondarily out of their presumed area of origin in the western part of the Irano-Turanian floristic region^19^.

The Altai Mountains are known as a refuge, e.g. for cold-adapted species during warm humid phases^87, 88^, and could have served as a refugium for steppe and semi-desert plants such as *K. ceratoides* during the interglacial phases of the Quaternary. As a consequence, the Altai Mountains could have been a source for recolonisation of the spreading steppe area during glacial periods. Because of the numerous large populations of *K. ceratoides* in Central Asia with high morphological variation in leaf size and shape, hair density, and plant height, this area was assumed to be the cradle of the species^35, 46, 49, 89–96^. Our findings suggest western Central Asia and the Altai Mountains as the area of origin for *Krascheninnikovia* (Fig. 7) and the starting point of its range expansion throughout the Northern Hemisphere. As indicated by their high private allelic richness, the populations of eastern and western Central Asia harbour a diverse gene pool (Supplementary Figure S5). Thus, these may have served as a good source for dry steppe plants for recolonising the surrounding areas after times of unsuitable, more humid conditions^97^ during the interglacial periods.

Although the stem lineage of *Krascheninnikovia* may have existed since the Pliocene^34, 49, 50^, it has only diversified in the last ~ 2.2 My^35^, coincidental with the beginning of the Pleistocene glacial-interglacial macro cycles. It seems that the cold, dry climate during the glacial stages facilitated the migration of *Krascheninnikovia*, as opposed to the more humid and less continental climatic conditions earlier during the Neogene^1^.

*Krascheninnikovia ceratoides* shows an apparent isolation-by-distance pattern: populations that are geographically close are usually genetically similar (Figs. 2, 5, 6, 7). The populations of Central Asia, which belong to two distinct genetic lineages, are exceptions. While geographically close to the populations of western Central Asia and separated only by the Khangai Mountains, the eastern populations are genetically more similar to the populations of North America. The Khangai Mountains (covered by alpine vegetation, taiga, mountain steppe, and forest-steppe^98^) have divided the populations east and west of the mountains and prevented gene flow for a long time. This split occurred in the early stages of diversification, after *Krascheninnikovia* migrated from the Altai Mountains to eastern Central Asia (Fig. 7).

A migration from eastern Central Asia to North America as hypothesised by Heklau and Röser^23^ and Seidl et al.^35^ is here confirmed. The dispersal to North America may have occurred across Beringia, which repeatedly connected the Asian and American continents during several arid glacial phases in the Pleistocene^99, 100^.

The sister group relationship of populations in North America (USA) on the one hand and eastern Central Asia (East Mongolia) on the other was found in all phylogenetic inferences (Figs. 3, 4) as well as in Seidl et al.^35^ The two groups form strongly supported clades (Figs. 2, 3, 4, 6, Supplementary Figure S4), which hints at low or absence of gene flow.

Starting from its likely area of origin in western Central Asia and the adjacent Altai mountains, *Krascheninnikovia* migrated west to Middle Asia.

During the Middle Pleistocene, glaciers dammed the Siberian Ob-Irtysh-Tobol river system, leading to backwaters that reached the foothills of the Kazakhstan Highlands and the Altai Mountains. The resulting landscape of lakes and swampy areas was not a suitable habitat for steppe plants^1, 83, 85^. According to our biogeographic analysis, this probably also affected the phylogeographic patterns of *K. ceratoides*, which was probably present on the Kazakhstan plain and even further west at this time. This damming would have caused range splits and contractions, followed by isolation and subsequent expansion. Repeated secondary contact may be the explanation for the absence of monophyly for individuals from eastern Middle Asia in our IQ-TREE tree (Supplementary Figure S4). It has been shown that the histories of other steppe taxa such as *Clausia aprica* (Stephan) Korn.-Trotzky^12^, *Goniolimon speciosum* (L.) Boiss.^18^, *Capsella* Medik.^13^, *Allium cretaceum* N.Friesen & Seregin, and *A. montanostepposum* N.Friesen & Seregin^14^ were affected by these backwater events as well.

Our results suggest that *Krascheninnikovia* migrated stepwise from Central Asia to Middle Asia and to Europe. Then, the European populations were separated from populations east of the Urals (Figs. 2, 4, 7). This genetic split may be traced back to a migration barrier imposed by a transgression of the Caspian Sea like the Apsheron transgression ~ 1 Mya^1^. This suggests that the separated populations found refugia in the Ural mountains^17, 18^ and west of the Caspian Sea^18, 101, 102^ to survive the interglacial phases. The same spatio-temporal pattern was observed for the steppe plant *Camelina microcarpa* Andrz. ex DC., whose two major groups split about 1.2 ± 1 Mya^19^.

According to our data, Central Europe was colonised through stepping-stone-like migration from the Altai Mountains to Central Europe, rather than by long-distance dispersal. Stepping-stone migration to Central Europe would indicate that trees present during glacial periods may potentially have grown in patches, as opposed to a dense tree cover^103^, allowing the steppe and semi-desert plant *Krascheninnikovia* to migrate step by step.

Diploid and tetraploid populations of *Krascheninnikovia* coexist in most regions^48^. In Eurasia, both ploidy levels are present in eastern Europe, Middle Asia, southern Central Asia, and eastern Central Asia (Fig. 1, Supplementary Table S1)^34, 47^. Most diploid samples from eastern Middle Asia (P030, P070, P072, P269) and southern Central Asia (P237, P238) belong to the light green cluster in our LEA analysis, suggesting a common ancestry. The same cluster membership is shared by the diploid individuals from central Middle Asia (P034) and eastern Europe (P057, P062), which are admixed. The westward migration of *K. ceratoides* was possibly accomplished by diploid individuals, followed by (auto-)polyploidization in the newly colonised areas, a process which occurred several times independently as a response to climatic fluctuations in the Pleistocene^104^.

There is an ongoing discussion about potential subspecies and species in the genus *Krascheninnikovia*. While some argue that *Krascheninnikovia* is monospecific with only two subspecies *ceratoides* and *lanata*^22^, others distinguish several entities at the specific level, such as *K. arborescens*, *K. ewersmanniana,* and *K. compacta* from Middle and Central Asia^48, 87–90^. Our data did not include samples representing these potential taxa, except for two populations identified as *K. ewersmanniana* (P069 and P070). Therefore, this study is not suitable to conclude on this topic. The results of our analyses support however the recognition of subspecies *lanata* as defined by Heklau and Röser^23^, as the North American individuals form a clearly distinguishable genetic lineage. Our data did not support the samples identified as *K. ewersmanniana* to belong to a distinct clade, as they are nested at different positions among morphologically typical subsp. *ceratoides* specimens from eastern Middle Asia. However, to confirm this hypothesis thorough morphological studies are necessary. Thus, we concur with the conclusions of Heklau and Röser^23^, recognizing (at the moment) two subspecific entities, i.e. subsp. *ceratoides* and subsp. *lanata* within monotypic *Krascheninnikovia*.

Our analyses provide insights into the history of *Krascheninnikovia* and its connection to the history of the Eurasian steppe belt, confirming and refining previous findings using GBS. The observed phylogeographic patterns reveal the common history of *Krascheninnikovia* and the steppe belt: The *Krascheninnikovia* lineage represents an autochthonous steppe element that evolved in situ during the Miocene when steppe precursors made their first appearance in Central Asia. Additionally, the Caspian Sea transgressions and the ice-dammed backwaters of the Ob-Irtysh-Tobol river system during the Pleistocene left clear imprints on the population genetic structure that persist until today. Nevertheless, there is still much to be investigated: The inclusion of further samples from the southernmost part of the range in Eurasia and from North America in future GBS analyses of *Krascheninnikovia* could allow the narrowing down of the area of origin and conclusions to be drawn about the number of migration events to America that have taken place.

## Supplementary Information

Supplementary Information

## Data Availability

The raw data is available at the European Nucleotide Archive (accessions ERS5794424 to ERS5794612).
