# Supplementary material for: The phylogeographic history of Krascheninnikovia reflects the development of dry steppes and semi-deserts in Eurasia
Source: Sci Rep. 2021 Mar 23;11:6645. doi: 10.1038/s41598-021-85735-z (PMC7988158; doi:10.1038/s41598-021-85735-z)

The phylogeographic history of *Krascheninnikovia* reflects the development of dry steppes and semi-deserts in Eurasia

Anna Seidl[1*], Karin Tremetsberger[1], Simon Pfanzelt[2], Frank R. Blattner[2], Barbara Neuffer[3], Nikolai Friesen[4,5], Herbert Hurka[3], Alexander Shmakov[6], Batlai Oyuntsetseg[7], Anže Žerdoner Čalasan[3], Polina V. Vesselova[8], Karl-Georg Bernhardt[1]

[1]Institute of Botany, Department of Integrative Biology and Biodiversity Research, University of Natural Resources and Life Sciences, Vienna (BOKU), Gregor-Mendel-Straße 33, 1180 Vienna, Austria

[2]Experimental Taxonomy, Leibniz Institute of Plant Genetics and Crop Plant Research (IPK), Corrensstraße 3, 06466 Gatersleben, Germany

[3]School of Biology/Chemistry, Osnabrück University, Barbarastraße 11, 49076 Osnabrück, Germany

[4]Botanical Garden of the Osnabrück University, Albrechtstraße 29, 49076 Osnabrück, Germany

[5]I.M. Sechenov First Moscow State Medical University Ministry of Health of the Russian Federation, Department of Pharmaceutical and Natural Sciences, Izmailovsky Boulevard, 8, Moscow, 105043, Russia

[6]South Siberian Botanical Garden, Altai State University, Lenina 61, 656049 Barnaul, Russia

[7]Department of Biology, School of Arts and Science, National University of Mongolia, University street 3, 14201 Ulaanbaatar, Mongolia

[8]Institute of Botany and Phytointroduction, Committee of Forestry and Wildlife, Ministry of Ecology, Geology and Natural Resources of the Republic of Kazakhstan, Timiryazeva Street 36D, 050040 Almaty, Kazakhstan

* Corresponding author

E-mail: anna.seidl@boku.ac.at

Keywords: Eurasian steppe belt; genotyping-by-sequencing; *Krascheninnikovia ceratoides*; phylogeography; Pleistocene cold steppe relict.

Supplementary Table 1. Localities and codes of the populations of *Krascheninnikovia ceratoides* and outgroups studied. The populations are grouped according to geographic-genetic groups as defined in this study. The DNA of the individuals belonging to the populations P207, P209, P210, P215 and P218 was extracted from herbarium material; therefore, the ploidy could not be measured. FC, measured by flow cytometry; S, ploidy inferred by software nquire and/or ploidyNGS; *N*, sample size for GBS; MD, missing data; D, diploid; T, tetraploid. Acronyms of herbaria: OSBU (University of Osnabrück, Osnabrück, Germany); WHB (University of Natural Resources and Life Sciences, Vienna, Vienna, Austria);

Taxon	Locality	Code	Ploidy: FC (*N*)	Ploidy: S (N)	*N*	Source: voucher number (collector(s); determiner)
**Outgroups**						
Axyris hybrida	Mongolia: Khövsgöl Province (48.601331° N 99.181319° E)	Ax	MD (1)	MD (1)	1	WHB 68600 (Bernhardt; Bernhardt)
Ceratocarpus arenarius	Russia: Rostow Oblast (47.333690° N, 43.954000° E)	P228	MD (3)	MD (3)	3	WHB 73141 (Pfanzelt; Pfanzelt)
***Krascheninnikovia ceratoides* subsp. *ceratoides*: Western Central Asia (WCAs)**						
K. ceratoides subsp. *ceratoides*	Mongolia: Bayan-Ölgii Province (48.933333° N, 89.800000° E)	P207	MD (1)	T (1)	1	OSBU 10608 (Neuffer; Oyuntsetseg)
K. ceratoides subsp. *ceratoides*	Mongolia: Bayan-Ölgii Province (49.050000° N, 89.733333° E)	P209	MD (1)	T (1)	1	OSBU 10359 (Neuffer; Oyuntsetseg)
K. ceratoides subsp. *ceratoides*	Mongolia: Gobi-Altai Province (45.874167° N, 98.102889° E)	P218	MD (1)	T (1)	1	OSBU 22540 (Neuffer et al.; Neuffer)
***Krascheninnikovia ceratoides* subsp. *ceratoides*: Southern Central Asia (SCAs)**						
K. ceratoides subsp. *ceratoides*	Mongolia: Gobi-Altai Province (45.007130° N, 93.257260° E)	P235	T (3)	T (5)	5	WHB 72467 (Oyuntsetseg; Oyuntsetseg)
K. ceratoides subsp. *ceratoides*	Mongolia: Gobi-Altai Province (45.587150° N, 93.440340° E)	P237	D (1)	D (5)	5	WHB 72472 (Oyuntsetseg; Oyuntsetseg)
K. ceratoides subsp. *ceratoides*	Mongolia: Gobi-Altai Province (45.952933° N, 94.271800° E)	P238	D (1)	D (5)	5	WHB 72470 (Oyuntsetseg; Oyuntsetseg)
***Krascheninnikovia ceratoides* subsp. *ceratoides*: Eastern Central Asia (ECAs)**						
K. ceratoides subsp. *ceratoides*	Mongolia: Töv Province (47.638944° N, 104.897639° E)	P093	D (2)	D (2)	2	WHB 71181 (Bernhardt et al.; Bernhardt)
K. ceratoides subsp. *ceratoides*	Mongolia: Khövsgöl Province (49.513056° N, 99.090250° E)	P094	D (5)	D (5)	5	WHB 71087 (Bernhardt et al.; Bernhardt)
K. ceratoides subsp. *ceratoides*	Mongolia: South Gobi Province (43.848056° N, 103.170556° E)	P210	MD (1)	T (1)	1	OSBU 11936 (Hurka; Jamsram)
***Krascheninnikovia ceratoides* subsp. *ceratoides*: Russian Altai Mountains (RusAlt)**						
K. ceratoides subsp. *ceratoides*	Russia: Altai Republic (50.071611° N, 88.412167° E)	P036	T (2)	T (5)	5	WHB 68953 (Neuffer et al.; Neuffer)
K. ceratoides subsp. *ceratoides*	Russia: Altai Republic (50.072778° N, 88.374444° E)	P241	T (1)	T (5)	5	WHB 72629 (Neuffer and Friesen; Neuffer)
K. ceratoides subsp. *ceratoides*	Russia: Altai Republic (49.993450° N, 88.805546° E)	P242	T (1)	T (5)	5	WHB 72632 (Neuffer and Friesen; Neuffer)
K. ceratoides subsp. *ceratoides*	Russia: Altai Republic (50.402260° N, 86.689237° E)	P243	T (1)	T (5)	5	WHB 72630 (Neuffer and Friesen; Neuffer)
***Krascheninnikovia ceratoides* subsp. *ceratoides*: Eastern Middle Asia (EMAs)**						
K. ceratoides subsp. *ceratoides*	Russia: Altai Krai (51.141306° N, 81.197472° E)	P030	D (2)	D (5)	5	OSBU 24701 (Neuffer et al.; Hurka)
K. ceratoides subsp. *ceratoides*	Kazakhstan: Almaty Region (43.927306° N, 78.503389° E)	P069	T (1)	T (5)	5	WHB 71443 (Friesen and Seidl; Friesen)
K. ceratoides subsp. *ceratoides*	Kazakhstan: Almaty Region (44.345417° N, 78.830694° E)	P070	D (1)	D (5)	5	WHB 71442 (Seidl; Friesen)
K. ceratoides subsp. *ceratoides*	Kazakhstan: East Kazakhstan Region (47.157389° N, 80.692917° E)	P072	D (5)	D (5)	5	WHB 71444 (Friesen and Seidl; Seidl)
K. ceratoides subsp. *ceratoides*	Kazakhstan: Almaty Region (45.813278° N, 80.367972° E)	P073	T (5)	T (5)	5	- (Friesen and Seidl; Friesen)

K. ceratoides subsp. *ceratoides*	Kazakhstan: Almaty Region (43.072083° N, 78.426278° E)	P074	T (5)	T (5)	5	WHB 71450 (Friesen; Seidl)
K. ceratoides subsp. *ceratoides*	Kazakhstan: Almaty Region (44.226444° N, 78.811944° E)	P076	T (4)	T (5)	5	WHB 71452 (Friesen and Seidl; Bernhardt)
K. ceratoides subsp. *ceratoides*	Kazakhstan: East Kazakhstan Region (47.968056° N, 83.268000° E)	P077	T (5)	T (5)	5	WHB 71453 (Friesen and Seidl; Seidl)
K. ceratoides subsp. *ceratoides*	Kazakhstan: Almaty Region (46.605472° N, 80.581139° E)	P079	T (1)	T (5)	5	WHB 71445 (Friesen and Seidl; Seidl)
K. ceratoides subsp. *ceratoides*	Kazakhstan: East Kazakhstan Region (47.303694° N, 84.847444° E)	P081	T (5)	T (5)	5	WHB 71457 (Friesen and Seidl; Seidl)
K. ceratoides subsp. *ceratoides*	Kazakhstan: East Kazakhstan Region (48.732361° N, 84.630556° E)	P269	D (5)	D (5)	5	WHB 76587 (Bernhardt and Seidl; Bernhardt and Seidl)
***Krascheninnikovia ceratoides* subsp. *ceratoides*: Southern Middle Asia (SMAs)**						
K. ceratoides subsp. *ceratoides*	Kyrgyzstan: Naryn (41.89831° N, 74.29642° E or 41.41817° N, 75.02267° E)	P025_D	D (1)	D (1)	5	WHB 64399 (Köttl; Köttl)
K. ceratoides subsp. *ceratoides*	Kyrgyzstan: Naryn (41.89831° N, 74.29642° E or 41.41817° N, 75.02267° E)	P025_T	MD (4)	T (4)	5	WHB 64399 (Köttl; Köttl)
K. ceratoides subsp. *ceratoides*	Tajikistan: Gorno-Badakhshan, Western Pamir (37.294167° N, 72.225833° E)	P067	T (4)	T (5)	5	WHB 71501 (Schönswetter; Schönswetter)
***Krascheninnikovia ceratoides* subsp. *ceratoides*: Central Middle Asia (CMAs)**						
K. ceratoides subsp. *ceratoides*	Kazakhstan: Qaraghandy Region (48.562028° N, 70.904444° E)	P032	T (5)	T (5)	5	OSBU 24791 (Hurka et al.; Neuffer)
K. ceratoides subsp. *ceratoides*	Kazakhstan: Qaraghandy Region (48.101139° N, 67.567139° E)	P033	T (1)	T (5)	5	OSBU 24909 (Hurka et al.; Neuffer)
K. ceratoides subsp. *ceratoides*	Kazakhstan: Qaraghandy Region (48.049750° N, 67.184417° E)	P034	D (1)	D (4), T (1)	5	OSBU 24893 (Hurka et al.; Neuffer)
***Krascheninnikovia ceratoides* subsp. *ceratoides*: Western Middle Asia (WMAs)**						
K. ceratoides subsp. *ceratoides*	Kazakhstan: Aktobe Region (50.143139° N, 54.730833° E)	P101	T (3)	T (5)	5	OSBU 25690 (Hurka et al.; Neuffer)
K. ceratoides subsp. *ceratoides*	Kazakhstan: Aktobe Region (48.857889° N, 59.208639° E)	P102	T (5)	T (5)	5	OSBU 25776 (Hurka et al.; Neuffer)
***Krascheninnikovia ceratoides* subsp. *ceratoides*: Eastern Europe (EEur)**						
K. ceratoides subsp. *ceratoides*	Russia: Orenburg Oblast (51.549028° N, 56.702278° E)	P057	D (1)	D (5)	5	WHB 71528 (Neuffer et al.; Neuffer)
K. ceratoides subsp. *ceratoides*	Kazakhstan: West Kazakhstan Region (50.861694° N, 53.178361° E)	P062	D (1)	D (1)	1	WHB 71523 (Neuffer et al.; Neuffer)
K. ceratoides subsp. *ceratoides*	Russia: Voronezh Oblast (49.927085° N, 40.772559° E)	P160	T (1)	T (5)	5	WHB 73014 (Seidl; Polujanov)
K. ceratoides subsp. *ceratoides*	Russia: Belgorod Oblast (50.759154° N, 37.945007° E)	P161	T (1)	T (5)	5	WHB 73013 (Seidl; Polujanov)
K. ceratoides subsp. *ceratoides*	Russia: Tatarstan (54.1° N, 53.3° E)	P215	MD (1)	T (1)	1	OSBU 7992 (Neuffer; Markov)
***Krascheninnikovia ceratoides* subsp. *ceratoides*: Central Europe (CEur)**						
K. ceratoides subsp. *ceratoides*	Romania: Cluj County (46.968139° N, 23.553611° E)	P026	T (2)	T (5)	5	WHB 64899 (Bernhardt; Bernhardt)
K. ceratoides subsp. *ceratoides*	Austria: Lower Austria, Oberschoderlee (48.641944° N, 16.347722° E)	P028	T (1)	T (5)	5	WHB 65005 (Bernhardt; Bernhardt)
K. ceratoides subsp. *ceratoides*	Austria: Lower Austria, Goggendorf (48.615000° N, 15.942139° E)	P029	T (4)	T (5)	5	WHB 65006 (Bernhardt; Bernhardt)
***Krascheninnikovia ceratoides* subsp. *lanata*: North America (NAm)**						
K. ceratoides subsp. *lanata*	USA: California (35.541667° N, 117.900000° W)	P039	D (1)	D (5)	5	- (Leitner; Leitner)
K. ceratoides subsp. *lanata*	USA: California (36.541667° N, 117.750000° W)	P040	D (5)	D (4), MD (1)	5	- (Leitner; Leitner)
K. ceratoides subsp. *lanata*	USA: Utah (40.781917° N, 112.785167° W)	P041	D (5)	D (5)	5	- (Leitner; Leitner)

Supplementary Table S2: Parameter test with reduced data set

16 populations (P026, P034, P040, P057, P062, P067, P073, P094, P160, P207, P209, P210, P215, P218, P237, P242) distributed over the entire sampling area were chosen, comprising 56 samples in total.

The three parameters m, M and n were tested. One parameter was altered per run and the others kept at default settings. The ranges for the parameters were 3 to 6 for m, 1 to 4 for M and 1 to 8 for n. The parameter value, for which the most variable loci present in at least 80% of all tested individuals were recovered, was chosen for the analysis with the complete data set.

Supplementary Figure S3. In LEA, 15 hypothetical ancestral populations (*K*) were tested with 100 repetitions each. According to the cross-entropy criterion implemented in LEA to determine the optimal number of clusters, data fit best to eleven distinguishable genetic lineages. However, it was decided to consider only eight clusters, since the cross-entropy values differ only little for *K*=8 to *K*=11. The interpretation of the general phylogeographic pattern is not affected.

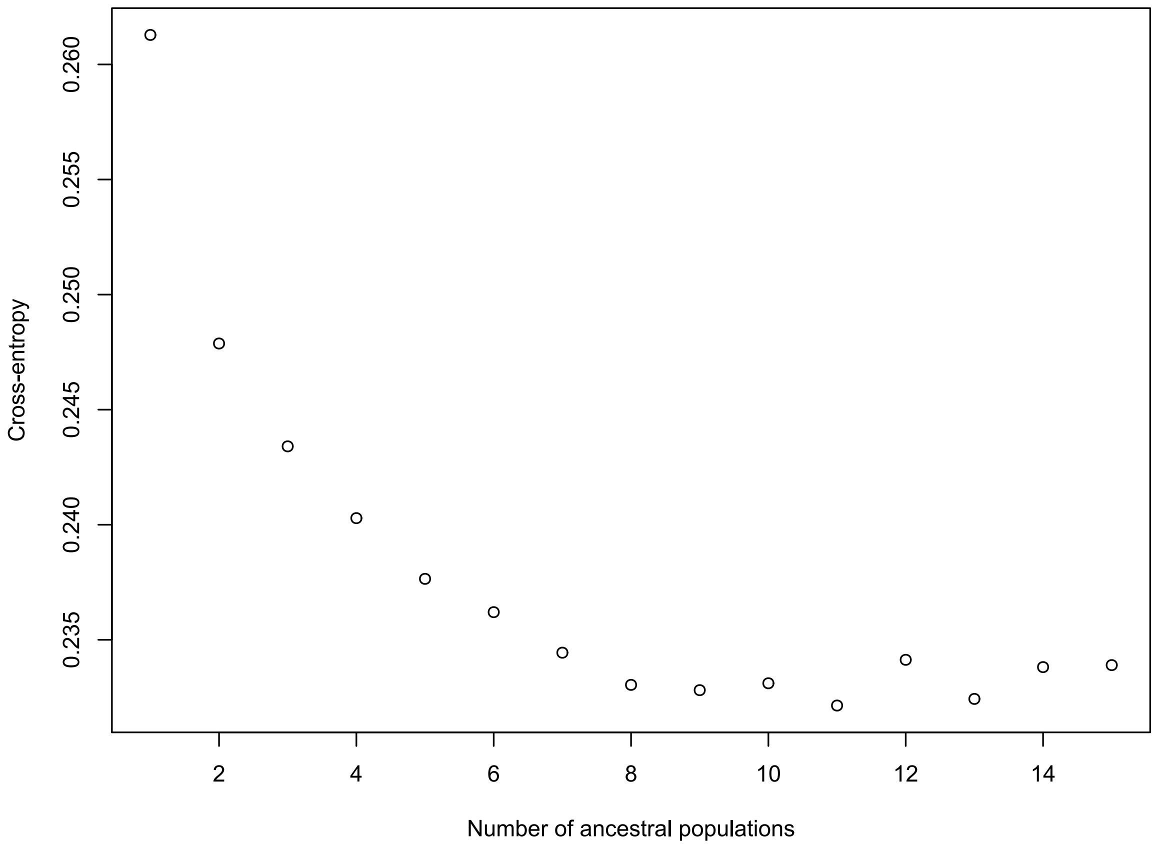

Supplementary Figure S4. Phylogenetic tree based on ML analysis of GBS data using IQ-TREE with GTR+G model and ascertainment bias correction. 1,000 repetitions of ultrafast bootstrapping (UFBoot) and of approximate likelihood-ratio test (aLRT) was performed. The support values are indicated at the nodes (SH-aLRT/bootstrap support value). Midpoint rooting was used. The origin of the samples is indicated by colour. The figure was visualized in FigTree v1.4.3 and edited in Inkscape v1.0.2 (https://inkscape.org).

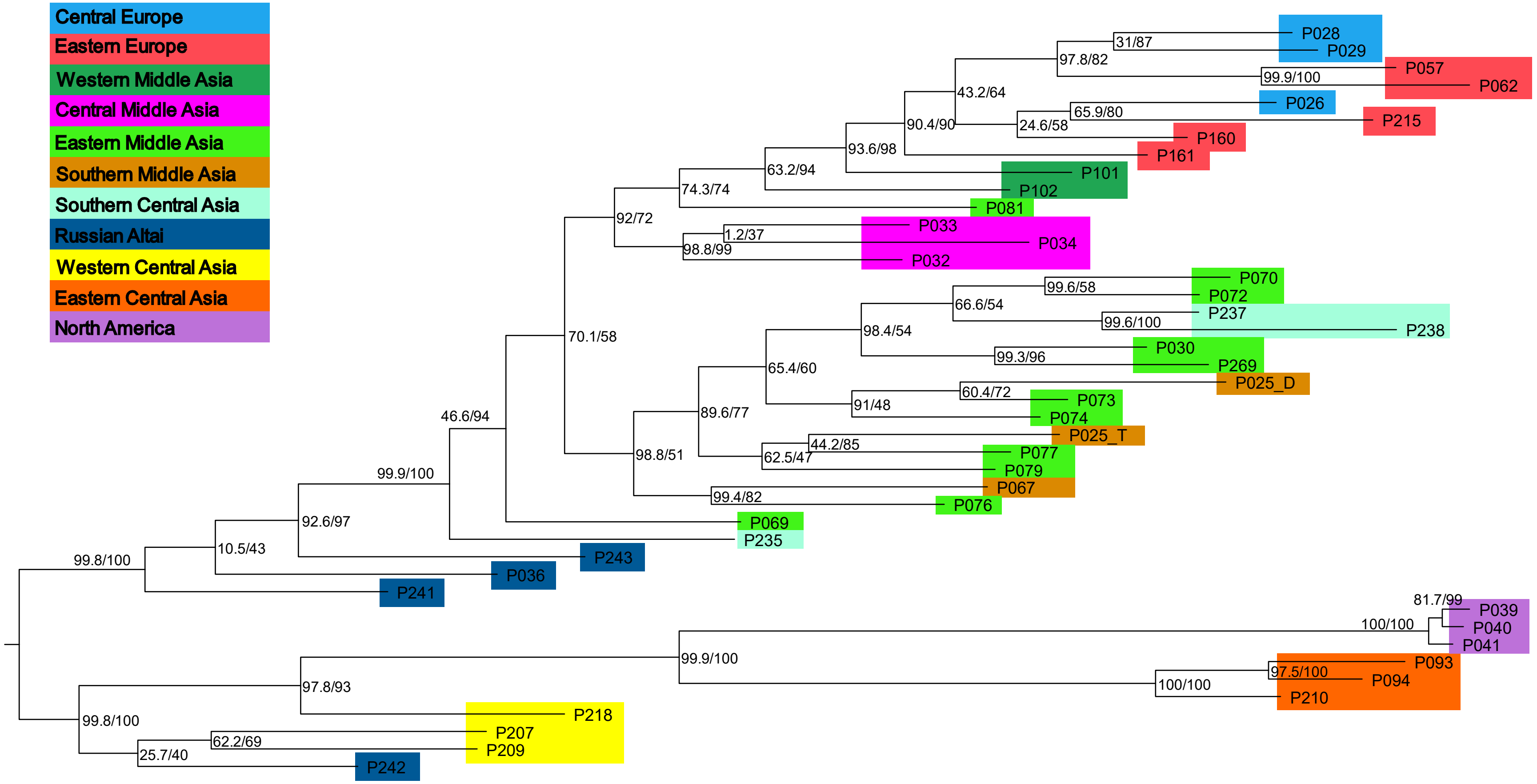

Supplementary Figure S5. Private allelic richness was computed for 1725 SNPs using HPrare. The highest private allelic richness was found in Central Asia.

Geographical region	Private allelic richness in geographic-genetic groups (average of population values)	SE
CEur	0.0090	0.0007
EEur	0.0090	0.0022
WMAs	0.0100	0.0020
CMAs	0.0105	0.0011
EMAs	0.0093	0.0021
SMAs	0.0092	0.0046
SCAs	0.0106	0.0022
RusAlt	0.0113	0.0009
WCAs	0.0144	0.0019
ECAs	0.0130	0.0038
NAm	0.0068	0.0029

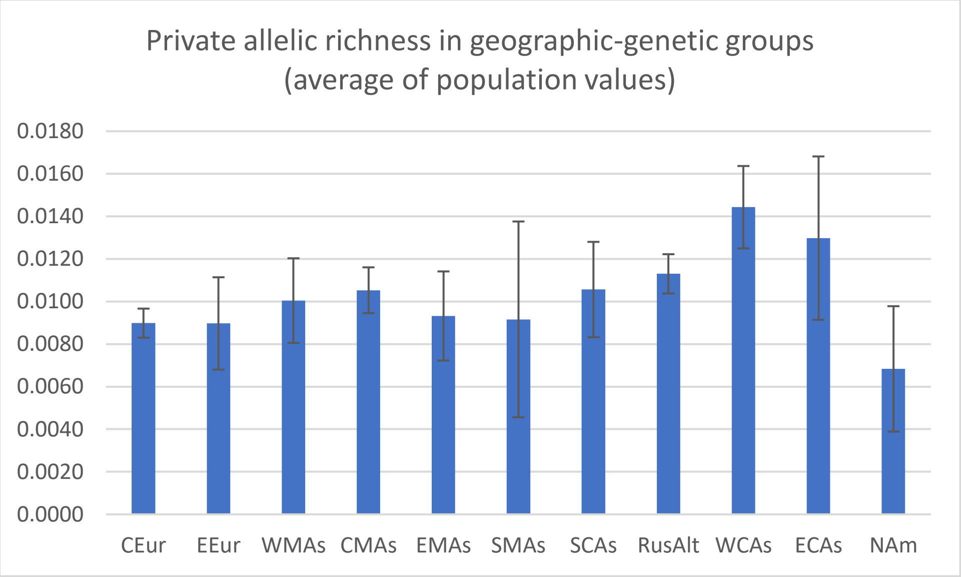

Supplement: Supplementary file 1 — Supplementary Information [file 41598_2021_85735_MOESM1_ESM.pdf]
